# 4-Amino­pyridinium picrate

**DOI:** 10.1107/S1600536810011050

**Published:** 2010-03-31

**Authors:** P. Ramesh, R. Akalya, A. Chandramohan, M. N. Ponnuswamy

**Affiliations:** a Centre of Advanced Study in Crystallography and Biophysics, University of Madras, Guindy Campus, Chennai 600 025, India; b Department of Chemistry, Sri Ramakrishna Mission Vidyalaya Arts and Science College, Coimbatore 641 020, India

## Abstract

In the title compound, C_5_H_7_N_2_^+^·C_6_H_2_N_3_O_7_^−^, the 4-amino­pyridinium cation is essentially planar (r.m.s. deviation = 0.002 Å). The three nitro groups in the picrate anion are twisted away from the attached benzene ring [dihedral angles = 24.1 (1), 9.3 (3) and 21.4 (1)°]. In the crystal structure, the ions are linked into a three-dimensional network by N—H⋯O and C—H⋯O hydrogen bonds.

## Related literature

For general background to picrate complexes, see: In *et al.* (1997 ▶); Zaderenko *et al.* (1997 ▶); Ashwell *et al.* (1995 ▶); Owen & White (1976 ▶); Shakir *et al.* (2009 ▶).
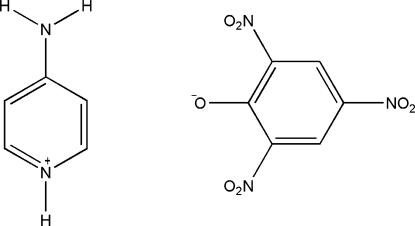


## Experimental

### 

#### Crystal data

C_5_H_7_N_2_^+^·C_6_H_2_N_3_O_7_^−^
*M* *_r_* = 323.23
Monoclinic, 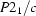

*a* = 8.5056 (7) Å
*b* = 11.3338 (9) Å
*c* = 14.3307 (11) Å
β = 104.162 (5)°
*V* = 1339.50 (18) Å^3^
*Z* = 4
Mo *K*α radiation
μ = 0.14 mm^−1^
*T* = 293 K
0.22 × 0.19 × 0.16 mm

#### Data collection

Bruker SMART APEXII area-detector diffractometer
Absorption correction: multi-scan (*SADABS*; Bruker, 2008 ▶) *T*_min_ = 0.970, *T*_max_ = 0.978
12562 measured reflections
3311 independent reflections
2637 reflections with *I* > 2σ(*I*)
*R*_int_ = 0.026

#### Refinement

*R*[*F* ^2^ > 2σ(*F* ^2^)] = 0.039
*wR*(*F* ^2^) = 0.108
*S* = 1.05
3311 reflections
221 parameters
H atoms treated by a mixture of independent and constrained refinement
Δρ_max_ = 0.24 e Å^−3^
Δρ_min_ = −0.18 e Å^−3^

### 

Data collection: *APEX2* (Bruker, 2008 ▶); cell refinement: *SAINT* (Bruker, 2008 ▶); data reduction: *SAINT*; program(s) used to solve structure: *SHELXS97* (Sheldrick, 2008 ▶); program(s) used to refine structure: *SHELXL97* (Sheldrick, 2008 ▶); molecular graphics: *ORTEP-3* (Farrugia, 1997 ▶); software used to prepare material for publication: *SHELXL97* and *PLATON* (Spek, 2009 ▶).

## Supplementary Material

Crystal structure: contains datablocks global, I. DOI: 10.1107/S1600536810011050/ci5057sup1.cif

Structure factors: contains datablocks I. DOI: 10.1107/S1600536810011050/ci5057Isup2.hkl

Additional supplementary materials:  crystallographic information; 3D view; checkCIF report

## Figures and Tables

**Table 1:** Hydrogen-bond geometry (Å, °)

*D*—H⋯*A*	*D*—H	H⋯*A*	*D*⋯*A*	*D*—H⋯*A*
N1—H1⋯O1^i^	0.91 (2)	1.82 (2)	2.6877 (16)	158 (2)
N1—H1⋯O7^i^	0.91 (2)	2.34 (2)	2.9359 (19)	122 (2)
N7—H7*A*⋯O6^ii^	0.88 (2)	2.30 (3)	3.139 (2)	160 (2)
N7—H7*B*⋯O5^iii^	0.88 (2)	2.23 (2)	3.065 (2)	158 (2)
C2—H2⋯O4^iv^	0.93	2.47	3.1373 (19)	129
C2—H2⋯O7^i^	0.93	2.43	2.9980 (19)	119

Symmetry codes: (i) 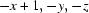; (ii) 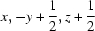; (iii) 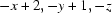; (iv) 
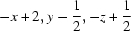
.

**Fig. 1.**
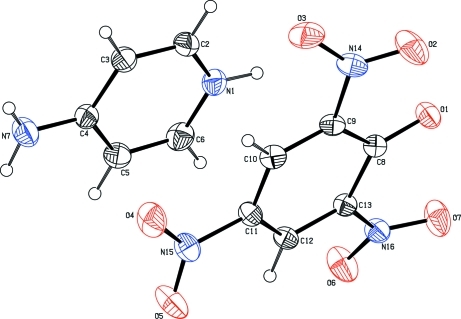
The asymmetric unit of the title compound. Displacement ellipsoids are drawn at the 50% probability level.

**Fig. 2.**
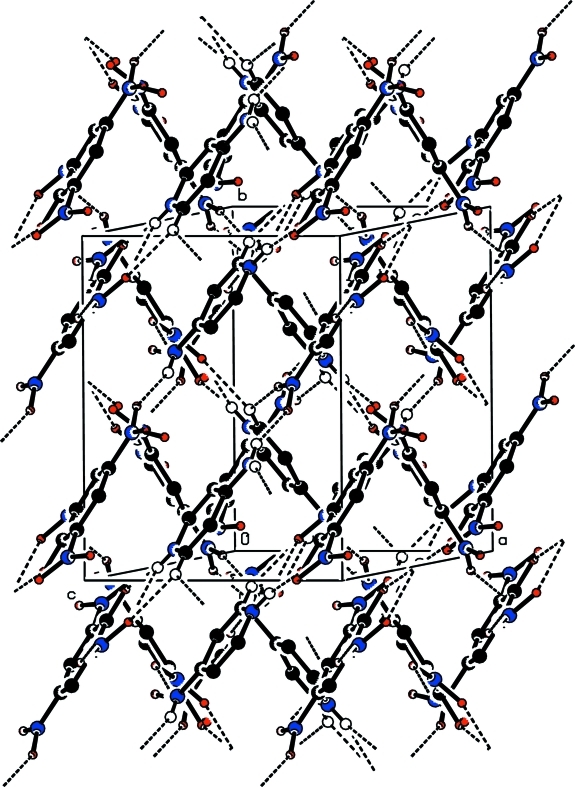
Crystal packing of the title compound. H atoms not involved in hydrogen bonding (dashed lines) have been omitted for clarity.

## supplementary crystallographic information

### Comment

It is well known that picric acid forms charge transfer molecular complexes with a number of aromatic compounds such as aromatic hydrocarbons and amines through electrostatic or hydrogen bonding interactions (In *et al.*, 1997; Zaderenko *et al.*, 1997). The bonding of donor-acceptor picric acid complexes strongly depends on the nature of partners. Some of the picric acid complexes crystallize in centrosymmetric space group though they possess non-linear optical (NLO) properties (Shakir *et al.*, 2009). This is due to the aggregation of the donor and acceptor molecules in a non-centrosymmetric manner which contribute to the bulk susceptibility from intermolecular charge transfer process (Ashwell *et al.*, 1995; Owen & White, 1976).

The 4-aminopyridinium cation is essentially planar (r.m.s. deviation 0.002 Å). In the picrate anion, as a result of deprotanation the C8—O1 distance [1.2392 (16) Å] shows a partial double bond character, and the C8—C9 [1.4568 (17) Å] and C8—C13 [1.4562 (17) Å] distances are longer compared to other aromatic C—C distances. The three nitro groups are twisted out of the attached benzene ring by 24.1 (1)° [N14/O2/O3], 9.3 (3)° [N15/O4/O5] and 21.4 (1)° [N16/O6/O7], which facilitate the interactions between the neighbouring molecules.

The ions are linked through N—H···O and C—H···O hydrogen bonds to form a three-dimensional network as shown in Fig. 2.

### Experimental

Equimolar solutions of 4-aminopyridine in methanol and picric acid in methanol were mixed together. The solution was stirred well for 1 h and the precipited salt was filtered off. The salt was repeatedly recrystallised from methanol to get single crystals suitable for X-ray analysis.

### Refinement

N-bound H atoms were located in a difference map and refined isotropically. C-bound H atoms were positioned geometrically (C–H = 0.93 Å) and allowed to ride on their parent atoms, with U_iso_(H) = 1.2 U_eq_(C).

### Crystal data

**Table:**

C_5_H_7_N_2_^+^·C_6_H_2_N_3_O_7_^−^	*F*(000) = 664
*M**_r_* = 323.23	*D*_x_ = 1.603 Mg m^−^^3^
Monoclinic, *P*2_1_/*c*	Mo *K*α radiation, λ = 0.71073 Å
Hall symbol: -P 2ybc	Cell parameters from 1853 reflections
*a* = 8.5056 (7) Å	θ = 2.3–28.4°
*b* = 11.3338 (9) Å	µ = 0.14 mm^−^^1^
*c* = 14.3307 (11) Å	*T* = 293 K
β = 104.162 (5)°	Block, colourless
*V* = 1339.50 (18) Å^3^	0.22 × 0.19 × 0.16 mm
*Z* = 4	

### Data collection

**Table:**

Bruker SMART APEXII area-detector diffractometer	3311 independent reflections
Radiation source: fine-focus sealed tube	2637 reflections with *I* > 2σ(*I*)
graphite	*R*_int_ = 0.026
ω and φ scans	θ_max_ = 28.4°, θ_min_ = 2.3°
Absorption correction: multi-scan (*SADABS*; Bruker, 2008)	*h* = −11→11
*T*_min_ = 0.970, *T*_max_ = 0.978	*k* = −15→14
12562 measured reflections	*l* = −18→19

### Refinement

**Table:**

Refinement on *F*^2^	Secondary atom site location: difference Fourier map
Least-squares matrix: full	Hydrogen site location: inferred from neighbouring sites
*R*[*F*^2^ > 2σ(*F*^2^)] = 0.039	H atoms treated by a mixture of independent and constrained refinement
*wR*(*F*^2^) = 0.108	*w* = 1/[σ^2^(*F*_o_^2^) + (0.0464*P*)^2^ + 0.3813*P*] where *P* = (*F*_o_^2^ + 2*F*_c_^2^)/3
*S* = 1.05	(Δ/σ)_max_ = 0.001
3311 reflections	Δρ_max_ = 0.24 e Å^−^^3^
221 parameters	Δρ_min_ = −0.17 e Å^−^^3^
0 restraints	Extinction correction: *SHELXL97* (Sheldrick, 2008), Fc^*^=kFc[1+0.001xFc^2^λ^3^/sin(2θ)]^-1/4^
Primary atom site location: structure-invariant direct methods	Extinction coefficient: 0.022 (2)

### Special details

**Table:**

Geometry. All esds (except the esd in the dihedral angle between two l.s. planes) are estimated using the full covariance matrix. The cell esds are taken into account individually in the estimation of esds in distances, angles and torsion angles; correlations between esds in cell parameters are only used when they are defined by crystal symmetry. An approximate (isotropic) treatment of cell esds is used for estimating esds involving l.s. planes.
Refinement. Refinement of *F*^2^ against ALL reflections. The weighted *R*-factor wR and goodness of fit *S* are based on *F*^2^, conventional *R*-factors *R* are based on *F*, with *F* set to zero for negative *F*^2^. The threshold expression of *F*^2^ > σ(*F*^2^) is used only for calculating *R*-factors(gt) etc. and is not relevant to the choice of reflections for refinement. *R*-factors based on *F*^2^ are statistically about twice as large as those based on *F*, and *R*- factors based on ALL data will be even larger.

### Fractional atomic coordinates and isotropic or equivalent isotropic displacement parameters (Å^2^)

**Table:**

	*x*	*y*	*z*	*U*_iso_*/*U*_eq_	
O1	0.87969 (13)	0.03133 (9)	0.06515 (7)	0.0445 (3)	
O2	0.97300 (19)	0.02415 (13)	0.25752 (9)	0.0734 (4)	
O3	1.20628 (16)	0.09976 (12)	0.31608 (8)	0.0632 (4)	
O4	1.35225 (16)	0.45326 (12)	0.18781 (9)	0.0664 (4)	
O5	1.23283 (19)	0.50978 (11)	0.04473 (10)	0.0700 (4)	
O6	0.92217 (17)	0.22433 (12)	−0.16703 (7)	0.0654 (4)	
O7	0.76429 (15)	0.11110 (13)	−0.11519 (8)	0.0686 (4)	
N1	0.37289 (15)	0.09339 (12)	0.03722 (9)	0.0441 (3)	
H1	0.293 (2)	0.0390 (18)	0.0159 (15)	0.068 (6)*	
C2	0.46638 (18)	0.09158 (14)	0.12753 (11)	0.0463 (4)	
H2	0.4515	0.0322	0.1694	0.056*	
C3	0.58135 (18)	0.17420 (14)	0.15875 (11)	0.0458 (4)	
H3	0.6451	0.1710	0.2215	0.055*	
C4	0.60534 (17)	0.26532 (13)	0.09690 (10)	0.0396 (3)	
C5	0.50530 (19)	0.26449 (15)	0.00273 (11)	0.0481 (4)	
H5	0.5164	0.3226	−0.0410	0.058*	
C6	0.39262 (19)	0.17818 (15)	−0.02361 (11)	0.0496 (4)	
H6	0.3271	0.1781	−0.0859	0.060*	
N7	0.71790 (19)	0.34716 (14)	0.12736 (12)	0.0543 (4)	
H7A	0.774 (3)	0.347 (2)	0.1874 (18)	0.083 (7)*	
H7B	0.721 (3)	0.404 (2)	0.0858 (16)	0.072 (6)*	
C8	0.96034 (15)	0.12354 (11)	0.07454 (9)	0.0325 (3)	
C9	1.06752 (16)	0.16121 (11)	0.16521 (9)	0.0338 (3)	
C10	1.15984 (17)	0.26074 (12)	0.17653 (9)	0.0364 (3)	
H10	1.2267	0.2793	0.2364	0.044*	
C11	1.15372 (17)	0.33411 (11)	0.09862 (9)	0.0356 (3)	
C12	1.05870 (16)	0.30622 (11)	0.00840 (9)	0.0346 (3)	
H12	1.0567	0.3554	−0.0438	0.041*	
C13	0.96746 (15)	0.20496 (11)	−0.00293 (8)	0.0327 (3)	
N14	1.08227 (17)	0.08920 (11)	0.25121 (8)	0.0442 (3)	
N15	1.25248 (16)	0.43901 (11)	0.11136 (9)	0.0455 (3)	
N16	0.87848 (15)	0.17853 (11)	−0.10042 (8)	0.0400 (3)	

### Atomic displacement parameters (Å^2^)

**Table:**

	*U*^11^	*U*^22^	*U*^33^	*U*^12^	*U*^13^	*U*^23^
O1	0.0511 (6)	0.0403 (6)	0.0398 (5)	−0.0122 (5)	0.0065 (4)	0.0028 (4)
O2	0.1011 (11)	0.0720 (9)	0.0468 (7)	−0.0287 (8)	0.0177 (7)	0.0152 (6)
O3	0.0769 (8)	0.0652 (8)	0.0380 (6)	0.0098 (6)	−0.0041 (6)	0.0119 (5)
O4	0.0691 (8)	0.0613 (8)	0.0600 (7)	−0.0231 (6)	−0.0010 (6)	−0.0163 (6)
O5	0.1005 (11)	0.0484 (7)	0.0610 (8)	−0.0287 (7)	0.0195 (7)	0.0038 (6)
O6	0.0881 (9)	0.0751 (8)	0.0285 (5)	−0.0328 (7)	0.0058 (5)	0.0052 (5)
O7	0.0656 (8)	0.0859 (9)	0.0437 (6)	−0.0384 (7)	−0.0070 (5)	0.0073 (6)
N1	0.0392 (6)	0.0432 (7)	0.0462 (7)	−0.0040 (6)	0.0036 (5)	−0.0038 (5)
C2	0.0447 (8)	0.0427 (8)	0.0476 (8)	−0.0033 (7)	0.0037 (6)	0.0092 (6)
C3	0.0437 (8)	0.0485 (9)	0.0393 (7)	−0.0047 (7)	−0.0010 (6)	0.0064 (6)
C4	0.0370 (7)	0.0388 (7)	0.0434 (7)	0.0002 (6)	0.0103 (6)	−0.0004 (6)
C5	0.0498 (8)	0.0520 (9)	0.0413 (8)	−0.0002 (7)	0.0090 (6)	0.0107 (7)
C6	0.0475 (8)	0.0611 (10)	0.0363 (7)	0.0000 (7)	0.0025 (6)	0.0014 (7)
N7	0.0582 (9)	0.0496 (8)	0.0526 (8)	−0.0150 (7)	0.0087 (7)	0.0019 (7)
C8	0.0353 (6)	0.0326 (6)	0.0303 (6)	0.0013 (5)	0.0090 (5)	−0.0008 (5)
C9	0.0420 (7)	0.0331 (6)	0.0267 (6)	0.0048 (5)	0.0094 (5)	0.0013 (5)
C10	0.0440 (7)	0.0366 (7)	0.0266 (6)	0.0026 (6)	0.0046 (5)	−0.0065 (5)
C11	0.0424 (7)	0.0304 (6)	0.0344 (6)	−0.0039 (5)	0.0103 (5)	−0.0060 (5)
C12	0.0431 (7)	0.0317 (6)	0.0296 (6)	−0.0004 (5)	0.0102 (5)	0.0006 (5)
C13	0.0366 (7)	0.0342 (6)	0.0260 (6)	−0.0004 (5)	0.0053 (5)	−0.0006 (5)
N14	0.0647 (8)	0.0376 (6)	0.0309 (6)	0.0053 (6)	0.0130 (5)	0.0027 (5)
N15	0.0547 (7)	0.0380 (6)	0.0451 (7)	−0.0098 (6)	0.0147 (6)	−0.0108 (5)
N16	0.0461 (7)	0.0396 (6)	0.0306 (5)	−0.0053 (5)	0.0021 (5)	0.0023 (5)

### Geometric parameters (Å, °)

**Table:**

O1—C8	1.2392 (16)	C5—C6	1.357 (2)
O2—N14	1.2069 (18)	C5—H5	0.93
O3—N14	1.2293 (17)	C6—H6	0.93
O4—N15	1.2214 (17)	N7—H7A	0.88 (2)
O5—N15	1.2267 (18)	N7—H7B	0.88 (2)
O6—N16	1.2216 (15)	C8—C13	1.4562 (17)
O7—N16	1.2130 (16)	C8—C9	1.4568 (17)
N1—C6	1.335 (2)	C9—C10	1.3613 (19)
N1—C2	1.3432 (19)	C9—N14	1.4578 (17)
N1—H1	0.91 (2)	C10—C11	1.3829 (19)
C2—C3	1.349 (2)	C10—H10	0.93
C2—H2	0.93	C11—C12	1.3832 (18)
C3—C4	1.408 (2)	C11—N15	1.4413 (17)
C3—H3	0.93	C12—C13	1.3726 (18)
C4—N7	1.328 (2)	C12—H12	0.93
C4—C5	1.408 (2)	C13—N16	1.4481 (16)
			
C6—N1—C2	120.05 (13)	C10—C9—C8	124.47 (11)
C6—N1—H1	118.0 (13)	C10—C9—N14	115.81 (12)
C2—N1—H1	121.9 (13)	C8—C9—N14	119.71 (12)
N1—C2—C3	121.28 (14)	C9—C10—C11	119.70 (12)
N1—C2—H2	119.4	C9—C10—H10	120.1
C3—C2—H2	119.4	C11—C10—H10	120.1
C2—C3—C4	120.36 (14)	C10—C11—C12	121.01 (12)
C2—C3—H3	119.8	C10—C11—N15	119.22 (12)
C4—C3—H3	119.8	C12—C11—N15	119.74 (12)
N7—C4—C3	120.62 (14)	C13—C12—C11	119.06 (12)
N7—C4—C5	122.52 (15)	C13—C12—H12	120.5
C3—C4—C5	116.85 (13)	C11—C12—H12	120.5
C6—C5—C4	119.43 (14)	C12—C13—N16	115.72 (11)
C6—C5—H5	120.3	C12—C13—C8	124.57 (11)
C4—C5—H5	120.3	N16—C13—C8	119.67 (11)
N1—C6—C5	122.02 (14)	O2—N14—O3	122.46 (13)
N1—C6—H6	119.0	O2—N14—C9	119.79 (13)
C5—C6—H6	119.0	O3—N14—C9	117.73 (13)
C4—N7—H7A	119.8 (16)	O4—N15—O5	122.97 (14)
C4—N7—H7B	115.0 (14)	O4—N15—C11	118.57 (13)
H7A—N7—H7B	125 (2)	O5—N15—C11	118.46 (13)
O1—C8—C13	125.23 (12)	O7—N16—O6	121.00 (12)
O1—C8—C9	123.57 (12)	O7—N16—C13	120.38 (11)
C13—C8—C9	111.14 (11)	O6—N16—C13	118.61 (11)
			
C6—N1—C2—C3	−0.1 (2)	C11—C12—C13—N16	−176.65 (12)
N1—C2—C3—C4	0.4 (2)	C11—C12—C13—C8	1.1 (2)
C2—C3—C4—N7	179.73 (16)	O1—C8—C13—C12	−179.30 (13)
C2—C3—C4—C5	−0.4 (2)	C9—C8—C13—C12	−2.13 (18)
N7—C4—C5—C6	−179.98 (16)	O1—C8—C13—N16	−1.6 (2)
C3—C4—C5—C6	0.2 (2)	C9—C8—C13—N16	175.54 (11)
C2—N1—C6—C5	−0.2 (2)	C10—C9—N14—O2	155.92 (14)
C4—C5—C6—N1	0.1 (2)	C8—C9—N14—O2	−24.9 (2)
O1—C8—C9—C10	178.44 (13)	C10—C9—N14—O3	−22.44 (18)
C13—C8—C9—C10	1.22 (18)	C8—C9—N14—O3	156.74 (13)
O1—C8—C9—N14	−0.7 (2)	C10—C11—N15—O4	8.1 (2)
C13—C8—C9—N14	−177.89 (11)	C12—C11—N15—O4	−169.71 (13)
C8—C9—C10—C11	0.7 (2)	C10—C11—N15—O5	−172.10 (14)
N14—C9—C10—C11	179.83 (12)	C12—C11—N15—O5	10.1 (2)
C9—C10—C11—C12	−1.9 (2)	C12—C13—N16—O7	−160.64 (14)
C9—C10—C11—N15	−179.76 (12)	C8—C13—N16—O7	21.5 (2)
C10—C11—C12—C13	1.1 (2)	C12—C13—N16—O6	20.26 (19)
N15—C11—C12—C13	178.88 (12)	C8—C13—N16—O6	−157.61 (14)

### Hydrogen-bond geometry (Å, °)

**Table:**

*D*—H···*A*	*D*—H	H···*A*	*D*···*A*	*D*—H···*A*
N1—H1···O1^i^	0.91 (2)	1.82 (2)	2.6877 (16)	158 (2)
N1—H1···O7^i^	0.91 (2)	2.34 (2)	2.9359 (19)	122 (2)
N7—H7A···O6^ii^	0.88 (2)	2.30 (3)	3.139 (2)	160 (2)
N7—H7B···O5^iii^	0.88 (2)	2.23 (2)	3.065 (2)	158 (2)
C2—H2···O4^iv^	0.93	2.47	3.1373 (19)	129
C2—H2···O7^i^	0.93	2.43	2.9980 (19)	119

Symmetry codes: (i) −*x*+1, −*y*, −*z*; (ii) *x*, −*y*+1/2, *z*+1/2; (iii) −*x*+2, −*y*+1, −*z*; (iv) −*x*+2, *y*−1/2, −*z*+1/2.

## References

[bb1] Ashwell, G. J., Jefferies, G., Hamilton, D. G., Lynch, D. E., Roberts, M. P. S., Bahra, G. S. & Brown, C. R. (1995). *Nature (London)*, **375**, 385–388.

[bb2] Bruker (2008). *APEX2*, *SAINT* and *SADABS* Bruker AXS Inc., Madison, Wisconsin, USA.

[bb3] Farrugia, L. J. (1997). *J. Appl. Cryst.* **30**, 565.

[bb4] In, Y., Nagata, H., Doi, M., Ishida, T. & Wakahara, A. (1997). *Acta Cryst.* C**53**, 367–369.

[bb5] Owen, J. R. & White, E. A. D. (1976). *J. Mater. Sci.* **11**, 2165–2169.

[bb6] Shakir, M., Kushwaha, S. K., Maurya, K. K., Arora, M. & Bhagavannarayana, G. (2009). *J. Cryst. Growth*, **311**, 3871–3875.

[bb7] Sheldrick, G. M. (2008). *Acta Cryst.* A**64**, 112–122. 10.1107/S0108767307043930 18156677

[bb8] Spek, A. L. (2009). *Acta Cryst.* D**65**, 148–155. 10.1107/S090744490804362X PMC2631630 19171970

[bb9] Zaderenko, P., Gil, M. S., López, P., Ballesteros, P., Fonseca, I. & Albert, A. (1997). *Acta Cryst.* B**53**, 961–967.

